# Feasibility and Acceptability of a Mental Health Website for Adults With an Intellectual Disability: Qualitative Evaluation

**DOI:** 10.2196/12958

**Published:** 2019-03-28

**Authors:** Chloe Watfern, Chloe Heck, Chris Rule, Peter Baldwin, Katherine M Boydell

**Affiliations:** 1 Black Dog Institute Sydney Australia; 2 University of New South Wales Art & Design Sydney Australia

**Keywords:** mental health, internet, intellectual disability, qualitative evaluation, qualitative research, health services accessibility

## Abstract

**Background:**

Electronic mental health (e-mental health) programs for people with an intellectual disability are currently underexplored but may provide a way of mitigating some of the barriers that this population faces in accessing appropriate mental health services.

**Objective:**

The aim of this study was to examine the feasibility and acceptability of Healthy Mind, an e-mental health program for adults with an intellectual disability developed by the Black Dog Institute, focusing on the design and implementation of the website.

**Methods:**

A qualitative research design was used, which involved semistructured interviews and focus groups with people with an intellectual disability, support workers, and allied health professionals. People with an intellectual disability were also observed while using the website. A thematic analysis was used to interrogate the interview transcripts and observational field notes.

**Results:**

Participants found the content of the website informative and appreciated the many ways that the website had been made accessible to users. Participants voiced some differing requirements regarding the way information should be presented and accessed on the website. Acknowledging different types of support needs was identified as an important issue for website dissemination.

**Conclusions:**

The Healthy Mind website promises to provide an excellent tool for people with ID and their supporters. This research has pragmatic implications for the future development and implementation of the program, while contributing to knowledge in the broader fields of e-mental health and inclusive design for people with an intellectual disability.

## Introduction

### Background

As a clinical term, intellectual disability (ID) denotes a lifelong impairment of cognitive functions that is associated with difficulties in a range of domains including, for example, learning and communication [1]. Approximately 1% of the world population has an ID [2]. ID is highly heterogeneous and must be understood in terms of the degree of support and environmental barriers that a given individual experiences [3].

People with an ID are more likely to experience common mental health problems than the general population [4], although prevalence estimates vary [5]. Despite early concerns regarding the suitability of psychological therapies for people with an ID [6], a recent meta-analysis found that appropriately modified cognitive behavioral therapies (CBTs) were an effective form of treatment [7]

Nevertheless, individuals with an ID face many barriers to accessing mental health services, from a lack of appropriate services to issues with communication and diagnosis [8]. For instance, many people with an ID find face-to-face interactions with health care professionals daunting, with some individuals opting to avoid doctors and other allied professionals whenever possible [9]. Digital mental health platforms have the potential to mitigate some of these barriers by providing readily accessible tools for both communication and treatment [9,10]. A plethora of such electronic mental health (e-mental health) programs are available for the general population, with substantial evidence to support the clinical effectiveness of many of these programs [11-13]. Despite available guidelines for adapting therapies for people with an ID [14,15], few e-mental health programs have been specifically tailored for users with an ID.

A team in Ireland has recently developed a computerized CBT game called Pesky Gnats, specifically adapted for use by adults with ID in sessions with a trained psychologist. The game was effective in reducing anxiety symptoms compared with treatment as usual for participants in a recent randomized control trial [16]. Similarly, Vereenooghe et al [17,18] found that computerized cognitive awareness training for people with ID improved the ability to identify behaviors and feelings, which is 1 important element of CBT. These studies provide promising evidence that digital mental health interventions can be an effective form of treatment for people with ID.

At another level, in the field of electronic health, there is growing acknowledgement that the usability and accessibility of platforms is a crucial factor that must be accounted for when considering their value and impact [19-21]. For people with ID, digital technologies are sometimes inaccessible [22]. For example, many platforms are not designed to account for the needs of people with cognitive differences. Accessible or inclusive design of digital technologies aims to ensure that digital platforms are readily useable by people with different abilities [23]. The Web Content Accessibility Guidelines 2.1 provide a comprehensive overview of how different features of a website can be optimized to promote accessibility [24].

A small body of research has begun to examine how people with ID interact with digital platforms, adapting methods from user experience and usability research to evaluate the design of websites and mobile apps [25,26-29]. To date, no research has examined the design of e-mental health platforms for people with an ID. Nevertheless, Cooney et al’s [30] qualitative study of the experience of participants in the Pesky Gnats randomized controlled trial highlighted some elements of program design, from the use of images to the delivery of a workbook, that affected participants’ engagement with the program.

### Objectives

The aim of this study was to evaluate the feasibility and acceptability of Healthy Mind, an e-mental health program for adults with IDs, with a focus on the relationship between the design of the platform and how users engage with and understand its content. This study had a pragmatic goal of informing further development of the website, along with strategies for its dissemination and implementation. However, as there is little existing research in the field of e-mental health for people with ID, this study also aimed to contribute to the knowledge base, informing best practices for inclusive design in this domain.

## Methods

### Study Design

The qualitative research design drew upon the emerging methodology of realist evaluation, which is ideal for evaluating complex interventions. The realist philosophy is positioned between positivist and constructivist paradigms and seeks to answer the questions *What works for whom, in what circumstances and why?* In seeking to answer these questions, realist evaluation enabled us to reflect on 3 fundamental components of the website: context, mechanism, and outcome [31]. The realist theoretical framework methodologically embraces pluralism, while emphasizing the importance of understanding complex social structures and relations when evaluating a given intervention.

Interviews and focus groups following semistructured schedules were conducted with lived experience experts (LEEs), support workers, and allied health professionals (AHPs) with expertise in ID mental health. To gain a deeper understanding of how people with ID interact with the website, LEEs were asked to work through a series of activities on the website, whereas the researchers observed and recorded field notes [28].

LEEs also completed a basic demographic questionnaire to ascertain age, level of experience with technology, and help-seeking strategies.

In focus groups and interviews, all participants were asked to reflect on what they liked and did not like about the website: its content, design, and the process of using the site. They were asked to comment upon any challenges they might have faced or that they believed others might face, elements of the website that they found to be particularly beneficial or otherwise good, and how they imagined the website could be used in day-to-day life, including specific probes for degree of support and context of use. Support workers were also asked to comment on the process of supporting an individual to use the website, and both support workers and AHPs were asked to consider how the website may be integrated into existing services. As the schedule of questions was semistructured, all participants were provided ample leeway to bring their own interests and unique perspectives to the discussions. Audio recordings varied in length between 18 and 70 min (mean 45.25 min). The research received ethical approval from the University of New South Wales (UNSW) Human Research Ethics Committee (approval reference HC180204).

### The Website

The Healthy Mind website was developed over an iterative process of consultation with key stakeholders and LEEs. It is an adapted version of the Black Dog Institute’s e-mental health platform, myCompass [32]. The target population for Healthy Mind is people with mild to borderline ID who want to build better mental health. In total, 2 key myCompass activities were chosen on the basis of consultation with researchers at UNSW’s Department of Developmental Disability Neuropsychiatry (3DN) specializing in ID mental health, along with a community advisory group consisting of people with ID, carers, health administrators, special educators, and clinicians who deliver mental health services to people with ID. These modules were chosen because they provide an introduction to 2 different but complementary approaches to better mental health. Please see Multimedia Appendix 1 for indicative screenshots of the website.

In the *Relax, Breathe Easy* activity, users are guided through breathing exercises for relaxation and body visualizations to reduce muscle tension and stiffness. In the CBT-based *Tackling Unhelpful Thinking* activity, users are taught to identify unhelpful thoughts and thinking traps, they are also introduced to the concept of a thought diary and how it might help to tackle unhelpful thoughts.

The content and design of these modules were modified, enhanced, and reconfigured to best suit the needs of the target population. A psychologist with extensive experience in providing and developing mental health services for people with Autism Spectrum Disorder and ID worked on the clinical content of the modules to modify the activities, ensuring strategies were in line with clinical best practice for this audience. An Easy Read Translation expert from the UNSW Centre for Social Impact ensured that all the content was in an accessible form: All the content is delivered in Easy Read English with an option for listening to an audio version of the text; video content also complements some of the tasks.

Additional consultation was sought with LEEs and their support workers at 2 stages during the development of the modules. Focus groups were conducted with 3 LEEs and 2 support workers where they were shown static pages from the website in development and were asked to comment on the look, feel, and design of the website. At the final stage of the development process, a working prototype of the website was shown to 2 people with ID and their support workers for feedback as they worked through the activities. All feedback was incorporated into the development and refinement of the interface.

### Participants

In total, 36 people participated in this study. LEEs were identified and recruited by supporting disability service providers in Sydney. The selection criteria for LEE participants were a diagnosis of ID, familiarity with computers, the ability to read and understand simple onscreen text, and the capacity to give informed consent regarding research participation. In total, 13 LEEs participated in the study (see Table 1 for LEE participant characteristics). In total, 9 support workers who accompanied LEEs also consented to participate in the research. These support workers were always present with LEEs while they used the website, providing assistance at their discretion. The ratio of support workers to LEEs varied between sessions; while some LEEs had one-on-one support, during 1 session there was a single support worker accompanying 3 LEEs. LEEs and their support workers all participated in focus groups. There were a total of 5 focus groups with these participants, which ranged from 2 to 7 participants per group (mean 4). In each focus group, at least 1 support worker was present.

In total, 5 AHPs participated in the research: a psychiatrist with specialization in neuropsychiatry and ID mental health, a social worker in a specialist disability health team, a speech pathologist working with children and adults in the disability sector, a community mental health outreach occupational therapist with a background in disability support, and a forensic mental health and cognitive disability expert. The occupational therapist and forensic mental health expert met together with a researcher in a small focus group, whereas the other 3 AHPs participated in one-on-one interviews.

Initially, the research team also hoped to recruit carers or unpaid supporters to participate in the research. Responding to ongoing recruitment difficulties, an advisory group shared by 3DN and the Black Dog Institute with expertise in ID was invited to participate in a focus group discussion. In total, 9 individuals participated in this focus group, including a clinical psychologist, a carer, a self-advocate, 2 psychiatrists, an ID e-mental health project officer, and senior staff from ID health and advocacy organizations.

### Data Analysis

All audio recordings of interviews and focus groups were transcribed verbatim for analysis. The final dataset also included observational and reflective field notes and amounted to over 150 pages of textual data. Thematic analysis was used as *a method for identifying, analyzing, and reporting patterns (themes) within the data* [33,34]. A bottom-up or inductive, data-driven approach to analysis was taken, focusing on the interpretation of the explicit or surface meanings of the data. In such a way, the analysis primarily described and summarized participant’s perspectives—a process of *giving voice* [35]—rather than deconstructing or critiquing.

CW followed Braun and Clarke’s [33] steps for thematic analysis, first familiarizing herself with the data, then embarking upon an iterative process of coding for semantic content, then searching for, reviewing, and naming themes. KB also reviewed potential themes by checking for coherence with selected extracts from the original transcripts. Final theme names and definitions were developed by CW and KB in collaboration as the writing process evolved. Research rigor was established via team analysis, prolonged engagement with the subject matter, and reflexivity.

**Table 1:** Lived experience expert participants' demographic, technology use, and help-seeking characteristics (N=13).

Variable			Statistics
**Age (years)**			
	Mean (SD)		31 (10.4)
	Range		19-52
**Gender, n (%)**			
	Female		7 (54)
	Male		6 (46)
**Technology use, n (%)**			
	Owns a computer		12 (92.3)
	Owns a smartphone		10 (76.9)
	Uses the internet daily		10 (76.9)
	Has used a health app		1 (7.8)
**Help-seeking, n (%)**			
	Has seen a mental health professional		7 (53.8)
	**If experiencing a personal problem, would seek help**		
		From family or friend	7 (53.8)
		From mental health professional	3 (23.1)
		Online	2 (15.4)

## Results

### The User Experience

**Figure 1:**
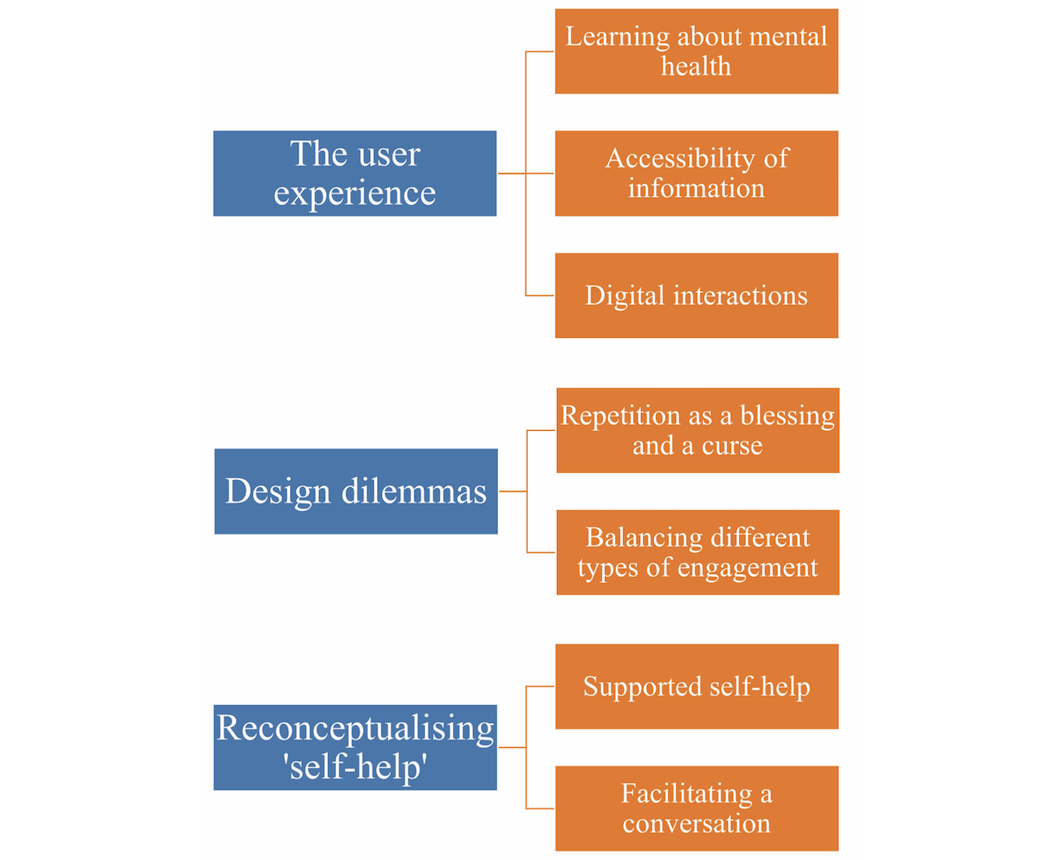
Thematic map.

We identified 3 overarching themes, subsuming 7 key themes, which are presented in a thematic map (Figure 1): The overarching theme *The user experience* comprises the themes *Learning about mental health, Accessibility of information*, and *Digital Interaction*; the overarching theme *Design Dilemmas* comprises the themes *Repetition as a blessing and a curse* and *Balancing different types of engagement*; and the overarching theme *Reconceptualizing self-help* comprises the themes *Supported self-help* and *Facilitating a conversation*.

### The User Experience

#### Learning About Mental Health

Many participants reflected that a key part of the experience of using the Healthy Mind website was learning about ways to promote and understand mental health. For instance, one LEE noted:

It helps me to think things and learn more about mental health.

Other LEEs also used the word *learn* to describe their experience:

I like to learn it;

I would love to listen with the voice again. Listen to the things again so I can gain more, so I can learn more.

LEEs commented on some of the specific skills gained while engaging with the website and how they made them feel. Many noted feeling more relaxed after doing breathing modules:

The breathing was good;

I do feel more relaxed;

5 big breaths, that’s all it takes.

Similarly, a support worker commented:

Whether it was because it took a lot of concentration or because it was doing its job incredibly well, there was a level of relaxation that fell over everyone and it was very quiet...I feel like it just had a very calming effect on people.

LEEs also commented on how the website might help with thinking and feelings:

It’s for your mind, your thinking, and it’s for your brains too. It’s good for the brains.

One LEE reflected on how the program had helped her to understand her own thoughts and feelings: "It allowed me to open up how I felt and why I felt like that the other day." However, she also noted that it might be tricky to use the skills in everyday situations: "It all depends on how angry and emotional you are." Overall, she recognized the value of the program but also wondered how she could ultimately use it to fundamentally change her mental health:

At the end of the day it’s something that is my experience and it’s something that is always probably going to be there and that I think I’m going to have to carry for the rest of my life.

#### Accessibility of Information

Participants provided feedback about the accessibility of information on the website, particularly considering the different types of needs that users may have when engaging with textual and audio-visual information. For instance, AHPs raised some concerns about the accessibility of the website for specific populations, including people with hearing and vision impairments; in its current form, the website is not adaptable to a screen-reader, and the video content is not captioned. AHPs also discussed accessibility issues for culturally and linguistically diverse users.

##### Easy Read

The use of Easy Read English was appreciated by LEEs:

I really liked it that it was very largely in easy read form.

I found that I was able to read the format easy because it had the pictures to explain.

AHPs also valued the use of short sentences accompanied by images. They were particularly positive about the use of *step-by-step* instructions.

Participants commented on the benefits of images within specific sections of the website as a way of elucidating the textual content, for example:

The image also shows you, it also gives you how you feel this.

LEE

However, LEEs also pointed out places where it would be difficult to understand the relationship between image and text or where people may have trouble interpreting images:

There were some pictures that I didn’t understand because it didn’t make sense and the pictures were the wrong pictures.

Some AHPs were concerned that users would have difficulty understanding different emotions using the current images on the website, particularly for people on the autism spectrum. Participants provided some conflicting opinions about the types of visual cues that would be helpful for users. Although some suggested using photographs of real human faces, others thought cartoon images or emojis would be more easily identifiable. One AHP suggested drawing upon similar imagery to the picture exchange communication system, which might already be familiar to users. AHPs also raised the issue of the tone of the images. One participant stated that it is important not to portray information “in a childlike manner.”

##### Audio and Video

During observations of website use, most LEEs accessed the audio function for some, if not all, of the textual content. Many gave positive feedback about the audio:

I liked the audio thing. It’s good, it helped me to understand more.

AHPs also noted that this audio function made the website more accessible to people who might not have a high degree of literacy. However, they also highlighted that the home page and some other parts of the website did not have this function, which could pose an access problem for some users.

The video content was appreciated by participants. During observations, all LEEs engaged with at least 1 video and gave feedback that they particularly enjoyed videos within the website. One LEE noted that “video is easier for me.” AHPs also responded positively to the videos as a means of promoting an understanding of the website content. Some recommended that videos may provide a more engaging way of introducing concepts or activities:

Definitely having more videos and interactive animation upfront before you go into all the detail.

#### Digital Interactions

##### Log In

Setting up a password and logging in to the website was a challenge for many LEEs, the majority of whom needed support to get through this process. Logging in seemed to cause anxiety in some users, who had their hands on their head, sighing, or making exclamations during this process. As a member of the advisory panel, the focus group said:

It’s basically asking people to do a whole bunch of arbitrary tests before they can actually—they might fail and it’s really understandable.

Remembering a password to log back in with was also a challenge for many LEEs. At another level, people might be deterred by the need to provide personal information during their first interaction with the site:

People would necessarily not feel comfortable with putting in their information unless they have probably had a familiarity with the website beforehand.

LEE

The log in process poses one of the biggest usability issues for this website. In its current state, it will likely deter many people from accessing the website, particularly if they are seeking to do so independently. However, design solutions need to balance multiple concerns that will be addressed in further sections of this paper.

##### Navigation

During observations, all LEEs could complete basic navigation tasks on the website, such as starting an activity, scrolling down the page, clicking next to move on to a new part of the activity, and clicking finish to finish an activity, although sometimes they needed prompting or extra assistance from a supporter. Many had difficulty navigating between parts of the second activity via the homepage dashboard, and participants suggested ways that the dashboard could be optimized so that it would be easier to navigate. In addition, participants suggested that progress within the activity should be made clearer, for instance, by including page numbers. Similarly, an LEE proposed:

How about instead of just saying next say what happens next in part so on and so on, that way they actually know that it’s a stepped activity program layout and if they don’t feel like doing it then they can press finish and come back to it when they need to.

##### Activities

During observations, many LEEs failed to complete some of the activities within the website despite clicking through the information on the page. For example, some participants did not hold their hand over their stomach to count breaths during the breathing activity or began deep breathing when the activity involved counting a normal breathing rate. Similarly, in a task on unhelpful thinking, participants did not answer questions asking them to reflect on how their thoughts made them feel. These signs of misunderstanding or not engaging with the activities could have been the result of any number of causes, not the least being the unnatural *testing* environment. The role of a support person in fostering engagement with the activities was critical, the implications of which are discussed further below. At another level, participants suggested that including more creative forms of interactivity and positive reinforcement within the website may increase motivation to engage with learning activities.

### Design Dilemmas

The current Healthy Mind website is designed so that certain information is repeated in a slightly varied form as a user progresses through the website. For example, information about how the website works is repeated at the beginning of each activity, but other types of information are also repeated, such as the idea of breathing to decrease stress. Participants offered conflicting responses regarding this repetition of content, which presents 1 design dilemma for the development team. On the one hand, some LEEs expressed frustration at points where they encountered information they had already seen:

OK, so some of the stuff is being repeated again. Like, I understand it but I got it the first time when I read it.

On the other hand, some LEEs seemed to appreciate the repetition and even wanted more: "Just recap the end...yeah, just more repeating." AHPs generally approved of the repetition of concepts within the site. For instance, an occupational therapist thought people should be encouraged to learn across multiple modalities:

How can you give the same information in a number of different formats so that you’re getting repetition and the option of taking it in in all the different ways that we learn?

The issue of repetition points to a larger issue of designing a single platform to meet the diverse needs of many different users who will have varying levels of literacy, receptive vocabulary, and other factors that affect the understanding of content. As one support worker commented:

It’s almost like different people will have different things that contradict each other, for instance the repetition could have been really helpful for someone else but...everyone is so different. That’s its own complication.

#### Balancing Different Types of Engagement

A distinct but related design dilemma that participants raised was the issue of how users may want to engage with the website. On the one hand, participants affirmed the importance of a linear, iterative, and personalized process of learning, where people could track their progress through the activities and return to complete them over time. Currently, the website is set up to create this kind of learning experience where users move progressively through the site at their own pace. An advisory group member commented on the importance of people being able to see how much they have completed of the program:

I think for people generally they really like a reinforcement. Either I’ve achieved something, or I’ve actually completed something.

This type of reinforcement is fostered by a personalized and step-by-step design.

On the other hand, participants described wanting something to access quickly for help in a crisis or to easily practice skills, for example, with video content or other simple tasks immediately at your fingertips:

It needs to be fast and quick, it needs to be like fast food, like a drive through...you need to be able to go and order and get your thing.

LEE

The analogy of the drive-through is an apt one when summarizing this kind of approach to website use, where many options are presented to the user who can quickly choose something that may help them at a given moment in time. A support worker similarly described the importance of having quick access to particular exercises:

Just say for instance one of the guys was on there and went on there because they were having a bit of a panic attack, maybe instead of having to go through everything, all the steps, if there was like an emergency button where they could click on it straight away and it would have breathing exercises and count downs to go through together with that person would be really good.

Here, she envisages using the website in response to an immediate need—for example, to calm someone down if they are experiencing a panic attack.

Other participants also mentioned ways of optimizing the home page so that users could more readily choose exercises that respond to how they are feeling in the moment:

Instead of all this stuff you have to get through to get to this, just say today I need something to help me to come down, breathing activities it is...and just having some simple go to apps on it or buttons.

AHP

Participants also suggested that this type of easy access to exercises would be a way of facilitating practice so that, for example, people could readily work through deep breathing on a daily basis.

Both quick and easy access to certain content, and a linear, accumulative learning experience could be contained within the same site. However, this would require rethinking the design of the website, particularly the homepage/dashboard and the log in process as it currently stands. As already discussed under the theme of digital interactions, requiring people to log in before accessing content creates an immediate hurdle to quick and easy access. However, if people use the site without logging in, then progress cannot be tracked and people will not be able to return to where they have left off during previous sessions. Furthermore, there are ethical dilemmas concerning interactive tasks where users provide personal information if the website is not password protected.

Many participants offered potential solutions to this predicament. Some thought that the log in should be completely discarded and the homepage reorganized so that users could choose between an array of exercises and simply remember where they were up to. Another recurring suggestion was that users’ first interactions with the site should not require logging in and that they should only be prompted to create a log-in profile once they proceed to activities that would benefit from collecting personal information and tracking progress.

### Reconceptualizing Self-Help

The Healthy Mind website is as an adapted version of the Black Dog Institute’s flagship e-mental health platform, myCompass, which has been described as *a personalized self-help tool for your mental health*. However, the notion of *self-help* needs to be clarified when considering the diverse support needs of people with an ID, as well as the way that this program may negotiate existing support systems.

#### Supported Self-Help

During the analytic process, the concept of *supported self-help* enabled the team to think through the ways that people may use this platform with the support of other people. A key finding during observations of website use was that when LEEs were working together closely with a support worker, they appeared more engaged with the tasks as they were able to talk over content and have assistance in completing activities. In contrast, many individuals who were largely unassisted in using the website were observed to be skipping through content very quickly or not actually completing the interactive tasks. AHPs also noted points in the program where they thought their clients would benefit from support in engaging with the activities. Support workers commented that some of the LEEs appeared overwhelmed or even had their anxiety triggered by not understanding what they were doing on the website. A sensitive supporter guiding someone through the website could perhaps help alleviate some of this anxiety of *not knowing*. For instance, a support worker conveyed the issues that she observed her clients facing as they attempted to negotiate the website by themselves, describing it as an “isolated experience...when you’re staring at a screen and being like overloaded with all of these words.” Instead, she advocated for a social approach to the tasks in a group setting, envisaging the website as a resource for support workers to guide their clients “so it all becomes more familial and more all-encompassing.”

Support workers expressed a tension between wanting to enable people to access the website independently as much as possible versus acknowledging and even encouraging people to engage with support as they use the site. For example, one support worker commented: “Sometimes you just want them to learn the way they want to learn.” This requires a certain degree of sensitivity on the part of a supporter to at once respect privacy and agency while ensuring that the person is enabled to fully understand the program. The support worker also commented: “[It] should be supportive for them until they really understand that website well.” Some users may find that they benefit from a trusted support worker showing them how to use the website and working through some exercises before going off and using it on their own. LEEs articulated differing views about whether they would like to be supported when using the website. Although some said they would want the help, others said:

I don’t mind using it by myself. I’m capable to do it myself, yeah.

One LEE with limited computer literacy did not have trouble understanding the website content but did require support to use the mouse, scroll down the page, and other basic navigational tasks. He commented: “You couldn’t do it by yourself if you didn't have any computer experience.” A *digital divide* does affect the ID population, with many people not having access to computer technologies and/or with limited computer literacy [22]. As one support worker said: “The barrier would be actually the computer literacy skills more than the website itself.” Acknowledging this divide, it is important that adequate support and resources are also in place to ensure that the website reaches people who may not be confident using computers.

Unfortunately, there is no clear-cut answer to how the tension between providing independence and support can be navigated. Ultimately, participants suggested that being clear about the many ways that people can use the website is of utmost importance—whether alone, with a friend, family member, or support worker. As one advisory group member commented: “I’m thinking is it a barrier if it’s set up as if you’re doing it on your own and someone’s feeling they need support to do it.”

#### Facilitating a Conversation

One unanticipated benefit of the website is its capacity to initiate communication between people that may not have occurred otherwise—in the words of an AHP: “this is a great way for a conversation to be facilitated.” Participants mentioned multiple ways that the website could operate within a broader social network of family members, support workers, and AHPs.

For example, the website was understood as a way of enabling users to discuss difficult to verbalize topics with people in their lives:

I think it’s a way of that support person getting an understanding as well of where that individual is. Because they might not be able to verbalise I feel really depressed...Even if they are not kind of strictly adhering to the modules, it’s that avenue towards communication that I think is really quite helpful.

AHP

In a similar way, the website was described as a safe space to “address the possible mental health concern without judgement” [AHP], which is particularly important for people who may be uncomfortable about identifying with either mental health problems or their disability and who may not seek out a counselor or other mental health professional. In this sense, the website was also understood as a potential gateway to other services or forms of help. For instance, an AHP described it as *a soft entry point*. Participants also recommended that the website could include examples of different words and phrases that people could use when they are asking for help—a way of building this process of facilitating conversation into the fabric of the platform. Others suggested that it include links to registered psychologists in the area or other services that may be helpful for mental health.

At another level, the website was frequently framed in response to a scarcity of existing resources—both a lack of training and expertise among AHPs and support workers and a lack of money and/or time to provide services. For example, an AHP commented on how the website might help with:

Things that I don’t have to do myself because I don’t have the capacity...this is—it wouldn’t replace kind of—but it’s a supplement.

Similarly, participants considered how the website could be used by supporters who do not have training in psychological interventions:

I think that could be filling a nice gap in there for people who maybe don’t have too much experience...working with clients on some of those like counselling sort of strategies.

AHP

Here, the website is framed as a tool that can help supplement existing services to provide better forms of psychological and behavioral support to clients.

## Discussion

This study employed a realist theoretical framework to explore the context, mechanism, and outcomes of the Healthy Mind website [31]. The context is that individuals with ID face barriers to access appropriate mental health services, and there is currently no research that examines the design of e-mental health platforms for people with ID. The mechanisms of website development were elucidated including the model of cocreation that involved people with ID and their networks of support. The outcomes were that website content was informative and accessible to users, although differing requirements vis-à-vis presentation of information were suggested. In addition, different support needs were identified, further clarifying the need to distinguish what works for whom and under what circumstances. The findings of this research have significance for the continuing development and dissemination of the Healthy Mind website, while at the same time presenting broader implications for the fields of e-mental health and inclusive design.

As in broader literature on inclusive or universal design, this study demonstrates that flexibility and multiple options for engagement lie at the core of truly accessible digital technologies [36]. Similar principles underpin many of the accessibility standards and guidelines; however, this study provides a uniquely situated insight into how different needs may be accommodated from the users’ perspective [37]. Further research could also consider how existing technology acceptance models may be used to evaluate and develop inclusive design projects such as Healthy Mind [38].

It was important to ensure that LEEs were enabled to provide feedback about the website; however, the literature provides few pointers regarding the best way to engage with people with ID in the design process. In general, guidelines suggested drawing upon multiple sources of evidence, including observations during use, while remaining responsive to the particular strengths and needs of participants [25,26,39]. Although some LEEs were very confident providing verbal feedback, during and after website use, others were much less so and this proved a significant challenge in a qualitative study requiring rich textual data. Nevertheless, this was mitigated by drawing upon observational notes and additional feedback from support workers who were present during testing. Similarly, consultations with AHPs and the advisory group added to the completeness of the picture.

However, additional work is certainly needed to develop more inclusive methods for engaging with people with ID in the design and evaluation process, particularly when verbal communication may be a challenge (these papers provide some interesting suggestions [40-42]). This is important for both research and intervention outcomes. For instance, involving users in the development of digital health products can lead to higher levels of engagement with the final platform [43]. However, the extent to which this applies for people with ID would be a fruitful avenue for further research.

The analysis generally treated all groups as a whole and did not attempt to compare and contrast between the perspectives of people with ID, support workers, and AHPs, instead identifying themes that were important in the data generated by all participant groups. In such a way, the points of contradiction in perspective that were identified during analysis occurred within all participant groups and not between them. For example, the contradiction between some participants wanting fast, easy access to resources and others emphasizing the importance of a progressive and step-by-step learning process was something that was highlighted by all participant groups. However, investigating the similarities and differences in perspectives between the different participant groups represented here would be an interesting avenue for future research. Considering potential points of difference in the perspectives of supporters and AHPs may also be important for website development, particularly when developing components of the website that are specifically tailored to supporters.

Despite some evidence of a digital divide affecting people with ID [22], the majority of LEEs did not have difficulties navigating the program or foresee issues with accessing a computer. The inclusion criteria for this study was that LEEs had a familiarity with computers, and the results of the survey show that all LEEs owned either a computer or smartphone and most used the internet on a daily basis, which may explain why the issue of a *digital divide* did not seem to be relevant to this group. Nevertheless, some AHPs and support workers did raise concerns about access to technologies for their clients. This study was limited by the fact that technology use and other demographic data were not collected routinely from the support workers and AHPs who participated in this study. For example, it is possible that these participants’ level of experience with technology may have influenced their perspectives. Regardless, it is important to consider how and where people will be able to use this program. For some, it may only be possible to use a computer in a communal or public space, or with the assistance of a supporter.

A key finding from this study is that the feasibility of the website ultimately depends on its ability to negotiate the different support needs and different systems of support that each user will bring to their engagement with the platform. Here, accessibility is reframed as something that can be fostered through both the design of the website *and* its dissemination—processes of knowledge translation and training will need to be developed to ensure that the website is accessible to people regardless of their support needs. Recent studies examining mobile assistive technology for people with ID also highlight the importance of training for users and/or the presence of ongoing support from a significant other [44,45]. Other research into e-mental health for people with ID has focused on the use of computerized therapy *with* psychologists [17,30]. When a platform such as Healthy Mind is intended to be used without the direct assistance of a counselor or psychologist, it is important to consider how linking in with other existing services may ensure that users are supported to engage with the program in a way that best suits their needs.

These findings can be considered in dialogue with broader literature surrounding e-mental health. E-mental health interventions have frequently been framed as a way of providing easy and free/inexpensive accessible services to people who may not otherwise choose to or be able to seek help from mental health professionals [11]. The Healthy Mind platform also seeks to serve this function. Interestingly, the idea that this website may also *facilitate a conversation* between people about mental health deepens our understanding of how e-mental health operates in society—for example, opening avenues to other types of help seeking [46]. Certainly, people with ID have unique support needs, which mean they may be more likely to use e-mental health programs with other people. However, the implications of this insight may also be relevant to a wider population of e-mental health users.

To conclude, this study provides a nuanced picture of the multiple and complex factors affecting how users engage with the Healthy Mind website. Acknowledging the importance of fostering engagement with e-mental health websites to increase their impact on well-being, this study highlights important issues that must be considered when designing and implementing e-mental health platforms for people with ID. The Healthy Mind website has the potential to provide an excellent tool for people with ID and their networks of support. We hope that this study will inspire further research into e-mental health programs such as this one.

## Appendix

Multimedia Appendix 1 Indicative screenshots of the Healthy Mind website.

## Abbreviations

AHP: allied health professional
CBT: cognitive behavioral therapy
e-mental health: electronic mental health
ID: intellectual disability
LEE: lived experience expert
UNSW: University of New South Wales
3DN: Department of Developmental Disability Neuropsychiatry
