# Supplementary material for: Feasibility and Acceptability of a Mental Health Website for Adults With an Intellectual Disability: Qualitative Evaluation
Source: JMIR Ment Health. 2019 Mar 28;6(3):e12958. doi: 10.2196/12958 (PMC6458530; doi:10.2196/12958)
Supplement: Multimedia Appendix 1 [file mental_v6i3e12958_app1.pdf]

Black Dog Institute

Healthy Mind - an Easy Read tool for building good mental health

Creating a mentally healthier world

Black Dog Institute

Healthy Mind is an online Easy Read tool for building good mental health

Start an Activity

Healthy Mind can help you feel better if you've felt

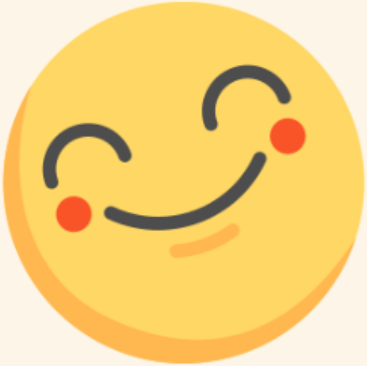

- worried for a long time
- stressed for long time
- angry for a long time
- sad for a long time

Healthy Mind will teach you how to think in ways that are helpful for having a good life

Activities

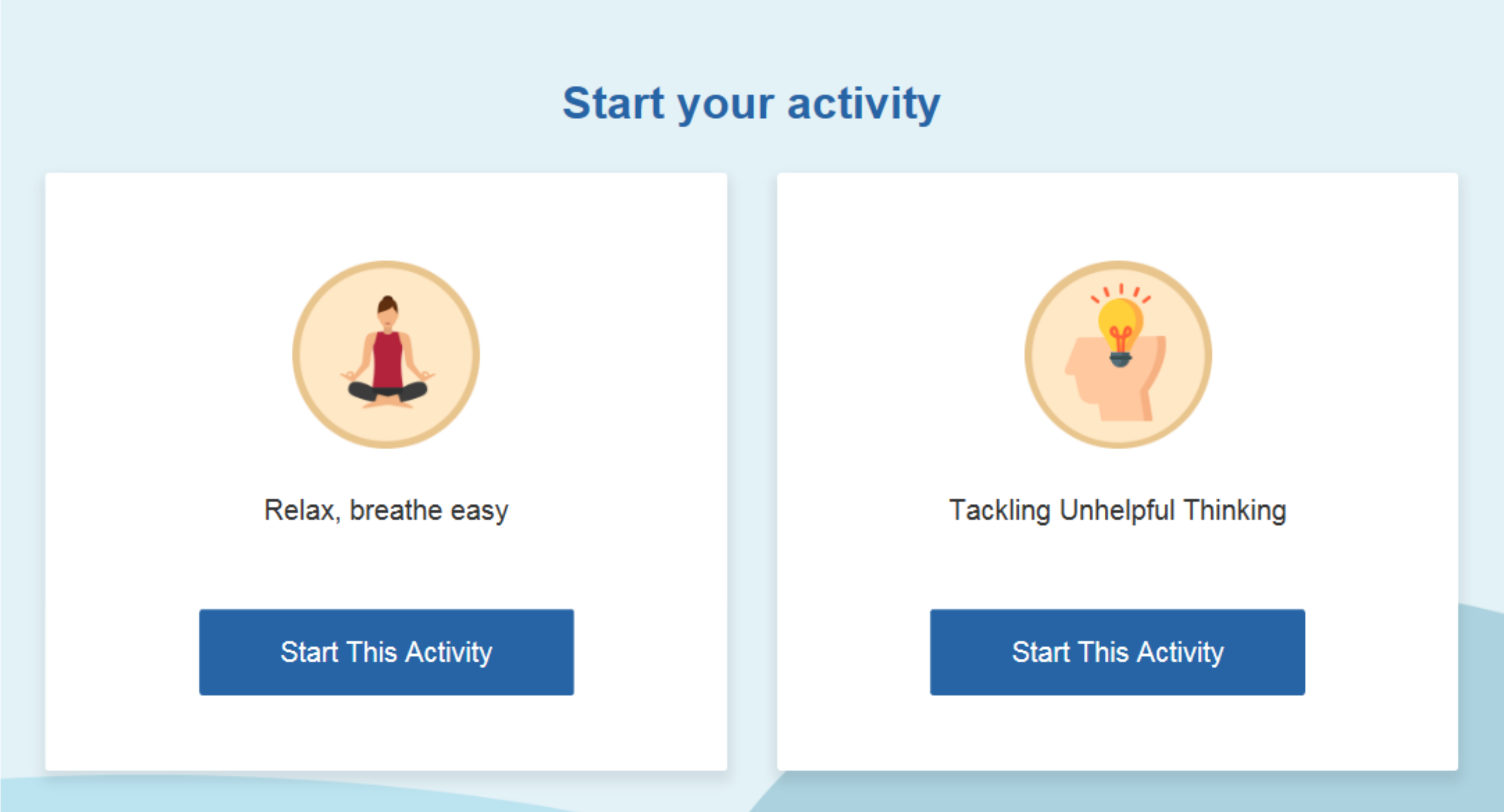
Start your activity
Relax, breathe easy
Start This Activity
Tackling Unhelpful Thinking
Start This Activity

Activities

What does it mean to be relaxed?

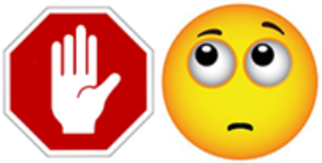

Being relaxed means that you stop feeling stressed or worried.

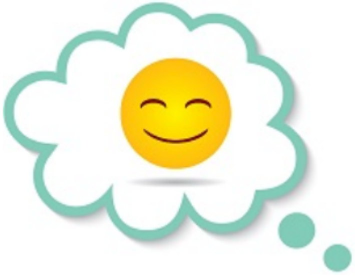

It means you can think about happy things.

Tracking progress

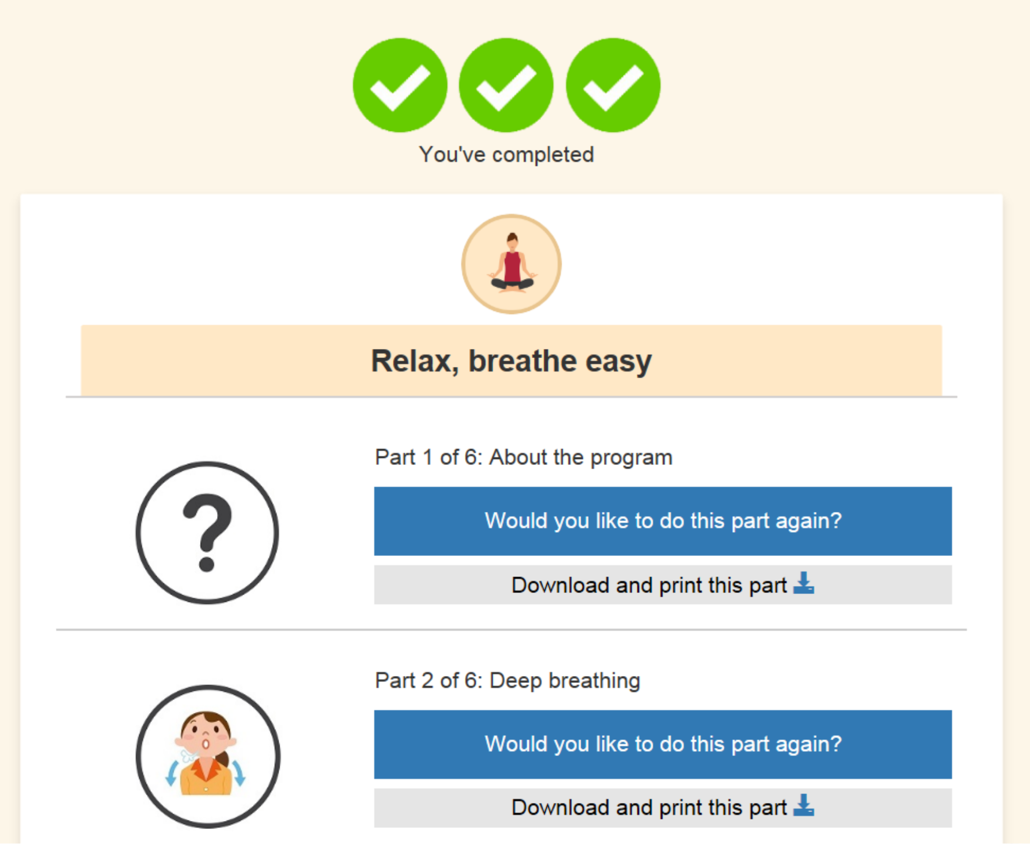

Videos

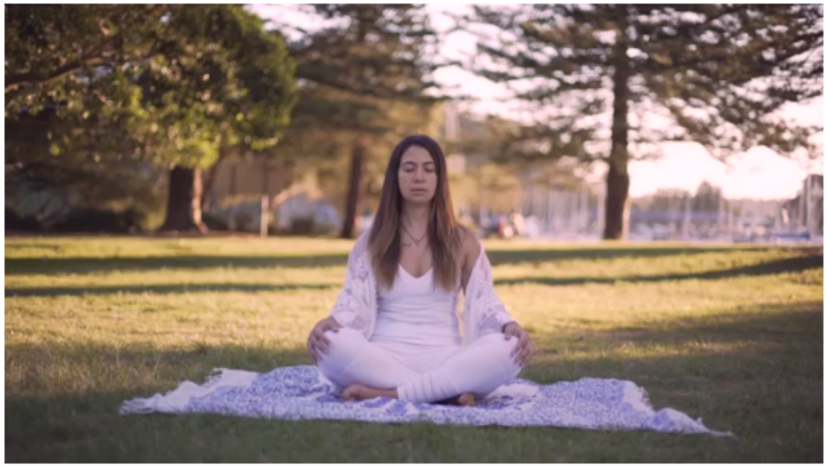

How to deep breathe

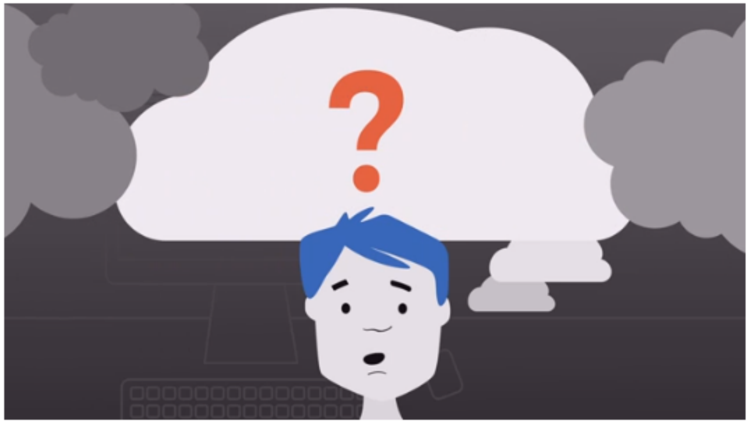

What is an unhelpful thought?

Log in

What is your first name?

What month and year were you born in?

--Select-- --Select--

Please select one of the following:

I don't have a phone number or email address

Please select a security question

--Select--

What's this for? We will ask you this question if you forget your password

Black Dog Institute

www.blackdoginstitute.org.au

/blackdoginst

Creating a mentally healthier world

Black Dog Institute
